# Identification, characterization and expression of novel Sex Hormone Binding Globulin alternative first exons in the human prostate

**DOI:** 10.1186/1471-2199-10-59

**Published:** 2009-06-17

**Authors:** Tomàs Pinós, Anna Barbosa-Desongles, Antoni Hurtado, Albert Santamaria-Martínez, Inés de Torres, Joan Morote, Jaume Reventós, Francina Munell

**Affiliations:** 1 Unitat de Recerca Biomèdica, Institut de Recerca Hospital Universitari Vall d'Hebrón, Barcelona, Spain; 2 Universitat Autònoma de Barcelona, Barcelona, Spain; 3 Servei d'Anatomia Patològica, Hospital Universitari Vall d'Hebrón, Barcelona, Spain; 4 Servei d'Urologia, Hospital Universitari Vall d'Hebrón, Barcelona, Spain

## Abstract

**Background:**

The human Sex Hormone Binding Globulin (SHBG) gene, located at 17p13.1, comprises, at least, two different transcription units regulated by two different promoters. The first transcription unit begins with the exon 1 sequence and is responsible for the production of plasma SHBG by the hepatocytes, while the second begins with an alternative exon 1 sequence, which replaces the exon 1 present in liver transcripts. Alternative exon 1 transcription and translation has only been demonstrated in the testis of transgenic mice containing an 11-kb human SHBG transgene and in the human testis. Our goal has been to further characterize the 5' end of the SHBG gene and analyze the presence of the SHBG alternative transcripts in human prostate tissue and derived cell lines.

**Results:**

Using a combination of *in silico *and *in vitro *studies, we have demonstrated that the SHBG gene, along with exon 1 and alternative exon 1 (renamed here exon 1A), contains four additional alternative first exons: the novel exons 1B, 1C, and 1E, and a previously identified exon 1N, which has been further characterized and renamed as exon 1D. We have shown that these four alternative first exons are all spliced to the same 3' splice site of SHBG exon 2, and that exon 1A and the novel exon 1B can be spliced to exon 1. We have also demonstrated the presence of SHBG transcripts beginning with exons 1B, 1C and 1D in prostate tissues and cell lines, as well as in several non-prostatic cell lines. Finally, the alignment of the SHBG mammalian sequences revealed that, while exons 1C, 1D and 1E are very well conserved phylogenetically through non-primate mammal species, exon 1B probably aroused in apes due to a single nucleotide change that generated a new 5' splice site in exon 1B.

**Conclusion:**

The identification of multiple transcription start sites (TSS) upstream of the annotated first exon of human SHBG, and the detection of the alternative transcripts in human prostate, concur with the prediction of the ENCODE (ENCyclopedia of DNA Elements) project, and suggest that the regulation of SHBG is much more complex than previously reported.

## Background

Sex hormone-binding globulin is a dimeric glycoprotein that transports sex steroids in the blood and regulates their access to target tissues [1]. Human SHBG gene is localized in the short arm of chromosome 17 (17p13.1), in a region characterized as hotspot for genetic recombination, gene amplification, and integration of foreign genomes [2,3]. In humans, the SHBG gene is constituted by a minimum of two different transcription units regulated by, at least, two different promoters [4]. The first transcription unit is responsible for the production of plasma SHBG by the hepatocytes, and begins with the exon 1 sequence, which encodes for a leucine rich signal secretion peptide. This transcription unit is regulated by the promoter 1 sequence that contains several binding sites for liver enriched transcription factors [4,5]. The second transcription unit begins with the alternative exon 1 sequence, which replaces the exon 1 present in the liver SHBG transcripts, and is regulated by an alternative promoter sequence [4,6], that proved to be very active when it was transfected in the GC2 mouse germ cell line [7]. The alternative exon 1 is found approximately 1.9 kb upstream of the exon 1 sequence, and does not contain an ATG in frame with the SHBG nucleotide coding sequence. It has been hypothesized that transcripts beginning with the alternative exon 1 could potentially initiate translation in the first ATG in frame found in exon 2, which encodes for the methionine 30 of the mature plasma protein [4,7].

The presence of SHBG mRNA has been demonstrated in human liver, brain, cardiac myocytes, adrenal glands, testis, prostate, mammary glands, placenta, fallopian tube, endometrium and granulose-lutein cells of the ovary [6,8-14]. However, transcription and translation of SHBG alternative exon 1 have only been shown in the testis of transgenic mice containing an 11-kb human SHBG transgene and in the human testis [6], resulting in a SHBG isoform that binds androgens and estradiol with high affinity, and accumulates in the acrosome of developing sperm [4].

In the prostate, the presence of SHBG mRNA and protein has been described in epithelial and stromal cells [15,16]. However, the transcription unit responsible for the synthesis of these SHBG mRNAs has not been characterized and it is unknown whether the protein found in the human prostatic tissues is translated locally or results from extravascularization of the liver secreted protein, as described in other tissues [17]. Our goal in the present study has been to identify novel SHBG TSSs since, as indicated by the ENCODE project, about two-thirds of the genes in the 1% of the analyzed human genome had unannotated 5' extensions [18], and determine which SHBG transcription units are active in human prostate.

## Methods

### Cell cultures

Human prostate cancer cell lines LNCaP, PC3, DU-145 and PZ-HPV7 were obtained from the American Type Culture Collection (ATCC, Rockville, MD). LNCaP, PC3 and DU-145 cells were maintained in RPMI 1640 medium (PAA Laboratories, Pasching, Austria), containing 10% fetal calf serum (PAA Laboratories), and supplemented with penicillin/streptomycin, sodium pyruvate and modified Eagle media with non-essential aminoacids as recommended. PZ-HPV-7 cells were grown in Keratinocyte-SFM Medium (Invitrogen, Carlsbad, CA), supplemented with 2.5 μg of EGF and 25 mg of bovine pituitary extract (both from Invitrogen). The hepatocarcinoma cell line HepG2 and the kidney carcinoma cell line Hek 293 were also obtained from ATCC and were grown and maintained in DMEM (PAA laboratories) containing 10% fetal calf serum, and also supplemented with penicillin/streptomycin, sodium pyruvate and modified Eagle media with non essential aminoacids.

### Human prostate samples

Human prostate samples were obtained from the non-tumoral tissue of patients with prostate carcinoma at the T2/T3N0M0 stage, submitted to radical prostatectomy. The informed consent was obtained in all cases, in keeping with Institutional Ethics Committee requirements. The histology of the prostate specimens was evaluated by the urological pathologist (Dr. de Torres).

### Bioinformatics

Using FirstEF: first exon and promoter prediction program for human DNA [19], we analyzed 900 nucleotides of the genomic sequence upstream of the characterized alternative exon 1 TSS, in order to identify potential SHBG 5' exons. To recognize potential exonic splicing enhancers of SHBG 5'exons, we used the combination of the following programs: RegRNA , ESEfinder 3.0 [20], and RESCUE-ESE [21]. To predict SHBG 5' exons secondary structure we used the MFOLD program [22].

### 5' Rapid Amplification of cDNA Ends (RACE)

5'RACE was performed using the FirstChoice^® ^RLM (RNA Ligase Mediated)-RACE Kit (Ambion, Austin, TX) with 10 μg of total RNA from DU-145 and LNCaP cells as template, and reverse primers complementary to the fifth (5' TGAGATCTCGGCCTGTTTGTC 3') and third (5' AGGCCTGCCGTCTCGAAGTCCC 3' for nested PCR) exon of SHBG gene. First round of PCR amplification consisted of 40 cycles of denaturation at 94°C for 20 sec, annealing at 59°C for 30 sec, and extension at 72°C for 45 sec. Nested PCR amplification was performed at the same conditions for 36 cycles. PCR products were cloned into the pCR^®^2.1-TOPO using the TOPO TA Cloning^® ^kit (Invitrogen). The inserts were amplified by PCR, purified with the QIAquick Gel Extraction Kit (Qiagen) and sequenced using an ABI Prism 3100 genetic analyzer (Perkin-Elmer Corp., Wellesley, MA).

### Identification of orthologous SHBG first exons in different species

To identify the orthologous sequences of alternative human SHBG first exons, we used the blastn, discontiguous megablast and megablast web interfaces of NCBI (expected threshold: 10; match/mismatch scores: 2,-3; gap costs: existence 5 extension 2), the NCBI Trace Archive  and the UCSC Genome Browser , and searched through the following database entries:*Pan troglodytes *[GenBank: NW_001226908.1] and NBCI trace id 268443681; *Gorilla gorilla *trace ids 1676028480, 1676347807, 1677542098, 2033098899; *Pongo pygmaeus *trace ids 892580595, 749280682; *Papio hamadryas *trace ids 1911889664, 1990873105, 1923887470, 257921900]; *Macaca mulatta *[GenBank: NW_001102932.1]; *Equus caballus *NCBI trace ids 1261023904, 1303970631, 1331950557, 1230075567; *Bos taurus *NCBI trace ids 666944906, 613752187; *Sus scrofa *NCBI trace id 863199273; *Canis familiaris *NCBI trace id 284698894; *Oryctolagus cuniculus *NCBI trace ids 1976086369, 1984382582; *Mus musculus *NCBI trace id 1092075609; *Rattus norvegicus *NCBI trace id 112313288. The recognized sequences were downloaded and aligned to the human SHBG sequences.

### RNA extraction, RT-PCR and Real-time PCR

Total RNA was isolated from LNCaP, PC3, PZ-HPV7, HepG2 and Hek 293 cell lines, and from human prostate tissue samples, using the RNeasy Mini/Midi Kit 50 (Qiagen, Hilden, Germany). Total RNA from rhabdomyosarcoma cell lines CW 9019 and RH 30, and from the neuroblastoma cell line *imr *32, was kindly provided by Dr. Josep Roma, and total RNA from the breast cancer cell lines MDA-MB 468, BT 474 and T47D was kindly provided by Dr. Maurizio Scaltriti. Two μg of RNA from each sample were reverse transcribed using Superscript II H^- ^(Invitrogen), at 42°C for 50 min. One μl of the resulting cDNA was amplified in a 25 μl reaction in the presence of Taq polymerase (Ecogen, Barcelona, Spain) or TaKaRa LA Taq™ (Takara Bio Inc., Shiga, Japan). The PCR amplification was performed in non-saturating conditions using the primer pairs described in Table 1. Each PCR was performed in triplicate. The PCR products were resolved by electrophoresis in a 1.5% agarose gel and purified, cloned and sequenced as described above.

**Table 1:** List of SHBG and s18 (control gene) primers used for RT-PCR

Target	Sequence	Annealing temp	Cycles
Exon 3 upper Exon 8 lower	5' CACGGCTGGATGATGGGA 3' 5' AGGGGGGTTCTTAGGTGGAGC 3'	59°C	39

Exon 1 upper Exon 5 lower	5' ATGGAGAGCAGAGGCCCACTG 3' 5' TGAGATCTCGGCCTGTTTGTC 3'	59°C	39

Exon 1A upper Exon 6 lower	5' TTCAAAGGCTCCCCCGCAGTGC 3' 5' GGAGACTGAGCCAAGATGGGT 3'	60°C	39

Exon 1B upper Exon 3 lower	5' TGAAGAGCCTGAGAGAGCG 3' 5' AGGCCTGCCGTCTCGAAGTCCC 3'	61°C	39

Exon 1B upper Exon 8 lower	5' TGAAGAGCCTGAGAGAGCG 3' 5' AGGGGGGTTCTTAGGTGGAGC 3'	59°C	39

Exon 1C upper Exon 3 lower	5' ACCTTCTTCACTGATCTTCAC 3' 5' AGGCCTGCCGTCTCGAAGTCCC 3'	57°C	39

Exon 1C upper Exon 5 lower	5' ACCTTCTTCACTGATCTTCAC 3' 5' TGAGATCTCGGCCTGTTTGTC 3'	58°C	35

Exon 1C upper Exon 8 lower	5' ACCTTCTTCACTGATCTTCAC 3' 5' TGGCTTCTGTTCAGGGCC 3'	58°C	39

Exon 1D upper Exon 3 lower	5' TTCCCAAAGGGACCGTGTG 3' 5' AGGCCTGCCGTCTCGAAGTCCC 3'	58°C	39

Exon 1E upper Exon 3 lower	5' CGTCGTAAATGGATTGACC 3' 5' AGGCCTGCCGTCTCGAAGTCCC 3'	57°C	39

S18 upper S18 lower	5' GATGGGCGGGGGAAAAT 3' 5' CTTGTACTGGCGTGGATTCTGC 3'	55–60°C	29

For real-time PCR, one μl of the cDNA was amplified in a 20 μl reaction using Quantitect™ SYBR^® ^Green PCR kit (Qiagen), with forward primers that recognized exons 1A, 1B, 1C and 1D and a common reverse primer for exon 3 (Table 1). The reactions were performed in triplicate, using the universal thermal cycling parameters (Applied Biosystems). Data were calculated as the means ± SE, for each SHBG alternative exon. The relative expression levels were calculated in relation to the levels of alternative exon 1A, according to the formula 2 ^-ΔCT^, where ΔCT is the difference in threshold cycle (CT) values between the target and the internal control (S18 gene) using one-way ANOVA.*P *values < 0.05 were considered significant.

## Results

### "In silico" identification of an additional SHBG first exon

**Figure 1:**
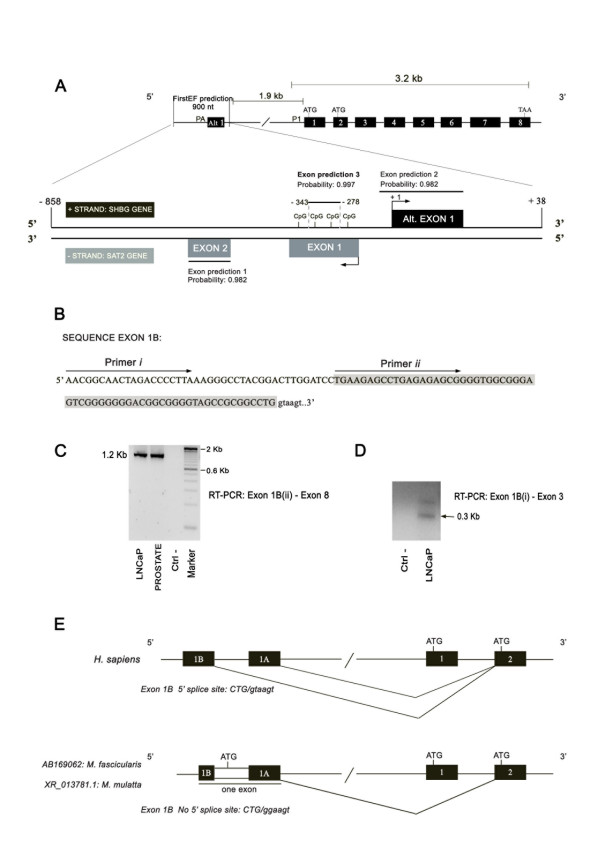
**Analysis of the annotated 5' end of the human SHBG gene**. A) The FirstEF program predicts three different sequences as potential first exons (exon predictions 1 to 3). The potential novel SHBG first exon is shown in bold print (exon prediction 3) and promoter 1 (P_1_) and alternative promoter (P_A_) are indicated. B) Exon 1B sequence (overlayed in gray) and primers (*i *and *ii*) (overlined) used for PCR amplification. C) RT-PCR amplification of the 1.2 kb exon 1B transcript in LNCaP cells and human prostatic tissue. D) RT-PCR amplification of the exon 1B transcript using primer (*i*). Arrow: expected 300 bp band. Upper band: sequence containing exons 1B-1-2-3. Ctrl-: negative control using water instead of cDNA. E) Comparison of the splicing patterns of exons 1A and 1B between humans and *Macaca*.

With the use of the FirstEF program, three potential first exons were predicted in the genomic sequence -858/+132 respective to the described alternative exon 1 TSS [23]: one in the negative strand of the chromosome 17 (7471406–7471590), with a genomic size of 185 nucleotides and a predicted exon probability of 0.982, that corresponded to the second exon of the SAT2 gene (exon prediction 1); and two in the positive strand in the same orientation of the SHBG gene (Figure 1A). Of the two predicted exons in the positive strand, one corresponded to the previously described alternative exon 1 (Figure 1A, exon prediction 2) [9,23], localized on the chromosome 17 genomic sequence 7471905–7472194, and with a predicted exon probability of 0.982; and the other corresponded to a putative previously non described SHBG first exon of 65 nucleotides in length, situated at -278/-343 respective to the alternative exon 1 TSS, and localized on the chromosome 17 genomic sequence 7471760–7471824 (Figure 1A, exon prediction 3). The predicted 3' end of this exon is perfectly defined by the consensus 5' splice site CTG/gtaagt (Figure 1B). According to the FirstEF program, the novel potential SHBG first exon is contained in a CpG island of 202 nucleotides in length localized on the chromosome 17 genomic sequence 7471654–7471855 (Figure 1A). We named this novel putative SHBG first exon, exon 1B, and therefore we renamed the SHBG alternative exon 1 as exon 1A.

In order to find out if exon 1B is expressed in prostate tissues and cell lines, we performed a PCR with a forward primer corresponding to the beginning of the predicted exon 1B (Figure 1B, primer *ii*), and a reverse primer of exon 8. Using the cDNA obtained from the LNCaP cell line and from human prostate samples, we amplified a major band of approximately 1200 bp (Figure 1C), which once cloned and sequenced, corresponded to the predicted exon 1B sequence followed by exons 2–3–4–5–6–7–8. The analysis of the sequence showed that all the exon-exon junctions resulted from splicing of consensus splice sites and that exon 1B transcripts used the same 3' splice site of exon 2 as exon 1 and exon 1A transcripts.

Aiming to determine whether exon 1B TSS was localized further 5' upstream of the sequence predicted by the FirstEF program, we designed three additional forward primers of exon 1B, situated -90, -70 and -39 nucleotides from the predicted exon 1B start site and performed PCR amplification using a reverse primer against exon 3 and cDNA from the LNCaP cell line. Using the primer situated -39 nucleotides from exon 1B predicted start site (Figure 1B, primer *i*), we obtained a major PCR product (Figure 1D), which, once sequenced, consisted of a 39 nucleotide extension of the exon 1B in its 5' end, followed by exons 2–3. This data allowed us to extend the exon 1B sequence to the nucleotide 7471721 of the chromosome 17, resulting for a characterized exon of 104 nucleotides in length (Table 2).

**Table 2:** Characteristics of human SHBG alternative first exons.

	Exon 1A	Exon 1B	Exon 1C	Exon 1D TSS1/TSS2	Exon 1D
Genomic position Chr 17	7472100–7472194	7471721–7471824	7471362–7471468	74758017/070–74758154	7457930–7458004

Strand	Positive	Positive	Positive	Positive	Positive

Length (nt)	95	104	107	137/85	75

Open reading frame	No (1uATG)	No	No	No (2/1uATG)	No (1uATG)

2ry structure (MFOLD) kcal/mol	-40.3	-64.4	-50.6	-45.3/-20.8	-35.8

5' splice site	TGG/gtaagc	CTG/gtaagt	TGG/gtacgg	AAG/gtatgt	GGG/gtgagt

Exonic splicing enhancers	SF2/ASF (2) SC35 (2) SRp40	SF2/ASF (5) SC35 (3) SRp55	SRp20 SRp40	SF2/ASF (2)/(0) SC35 (1)/(1) SRp40 (1)/(1) SRp55 (1)/(1)	SF2/ASF SRp40 (2) SRp55

To provide further evidence of the exon 1B expression, we analyzed the databases from other species and identified two mRNA sequences, one in *Macaca fascicularis *[GenBank: AB169062], and one in *Macaca mulatta *[GenBank: XR_013781.1]. The *Macaca fascicularis *mRNA sequence derived from a testis cDNA library and included an orthologous sequence of 36 nucleotides located upstream of the 5'splice site of the human exon 1B, and the *Macaca mulatta *mRNA sequence included the orthologous 21 nucleotides upstream of the 5'splice site in humans. Furthermore, in both *Macaca *mRNAs, the human 5' splice site was not found due to the presence of a GG sequence instead of the GT donor nucleotides of the human sequence, and therefore exon 1B and exon 1A are present in these mRNAs as one unique exon (Figure 1E).

### Identification of additional SHBG first exons using 5'RLM-RACE

**Figure 2:**
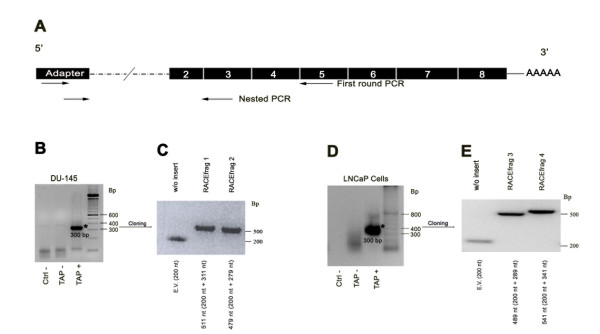
**5'RLM-RACE procedure in DU-145 and LNCaP prostate cancer cell lines**. A) Two forward primers used for the two rounds of RACE-PCR recognizing the adapter sequence, and two reverse primers, against the fifth and third exon of SHBG, are shown. B) In the DU-145 5' RACE, one major band (asterisk) was obtained. TAP+ samples include total RNAs treated with Tobacco Acid Pyrophosphatase, and TAP-samples are negative controls that include RNAs that did not incorporate the RNA Adapter oligonucleotide. Ctrl-: Negative controls performed using water instead of cDNA. C) Two cloned products were obtained from the major band: RACEfrag 1 and RACEfrag 2. The empty vector (E.V.) provided an amplification product of 200 nucleotides, corresponding to the M13 flanking sequence. D) In the LNCaP 5' RACE, one major band (asterisk) was obtained. E) The cloning of the major band generated RACEfrag 3 and RACEfrag 4 products. Insert and M13 flanking sequence size are indicated in the four RACEfrags.

**Figure 3:**
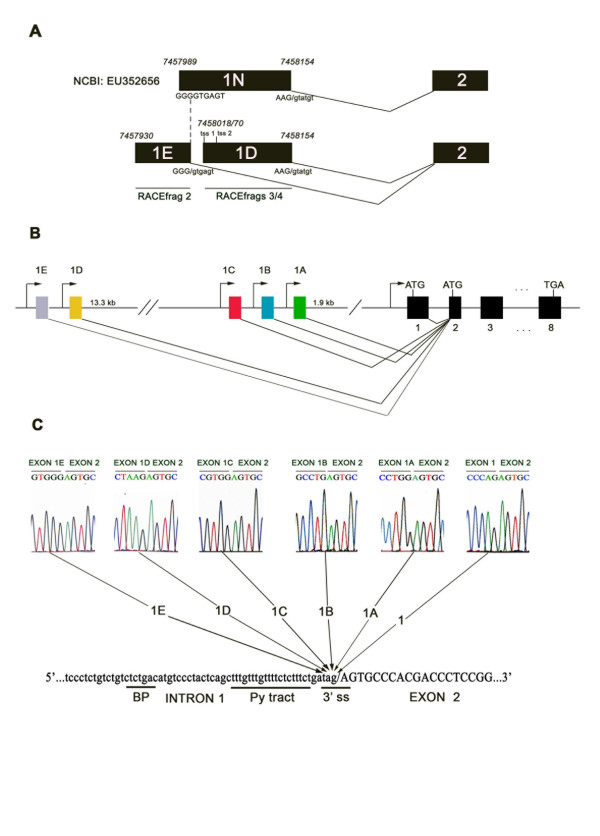
**Splicing patterns of the SHBG alternative first exons**. A) Comparison of the splicing pattern of the exon 1N with the exon 1E (RACEfrag 2) and exon 1D (RACEfrags 3 and 4). Numbers above the exons indicate the nucleotide position in the chromosome 17 of the TSSs (tss) and 3’ends of each exon. B) Diagram showing the 5’end of the SHBG gene. SHBG coding exons are shown as black boxes and alternative and non-coding SHBG first exons as coloured boxes. Arrowheads indicate the different SHBG TSSs. C) Electropherograms of SHBG transcripts showing the exon-exon junctions between the different alternative first exons and exon 2. All transcripts containing different alternative SHBG first exons use the same 3’splice site (3’ss) of exon 2. The consensus 3’splice site of intron 1, the pyrimidine tract (Py) and the potential branch point (BP) are shown.

To further characterize the 5' end of the SHBG gene, we used the FirstChoice^® ^RLM-RACE kit to identify 5'capped SHBG transcripts from total RNA of the DU-145 and LNCaP cell lines. To perform the first round of the 5' RACE, we used a forward outer primer that recognized the adapter sequence linked to the 5' end of capped mRNAs, and a reverse primer against exon 5 (Figure 2A). Nested PCR was performed using a forward inner primer of the adapter sequence and a reverse primer recognizing exon 3 (Figure 2A). In the DU-145 cell line, the two rounds of amplification resulted in one major product of 300 nucleotides approximately (Figure 2B), that, after cloning and sequencing, corresponded to two different and novel SHBG mRNAs. One RACE product (Figure 2C, RACEfrag 1; 311 nucleotides) contained a novel SHBG first exon of 107 nucleotides followed by exons 2–3, and the other RACE product (Figure 2C, RACEfrag 2; 279 nucleotides) consisted of a different and novel first exon of 75 nucleotides, also followed by exons 2–3. In the LNCaP cell line, the two rounds of amplification resulted also in one major band of 300 nucleotides approximately (Figure 2D), that after cloning and sequencing, corresponded to two different transcript sequences of 289 and 341 nucleotides (Figure 2E, RACEfrags 3 and 4). Both transcripts include an alternative SHBG first exon previously introduced in the public databases by Kahn and collaborators as exon 1N [GenBank: EU352656], followed by exons 2 and 3. However, these two RACEfrags contain two different TSS in their 5' end, resulting in an exon of 137 nucleotides when TSS 1 (Chr 17: 7458018) is used, and an exon of 87 nucleotides when the transcription starts at TSS 2 (Chr 17: 7458070) (Figure 3A). These sequences are also 27 and 79 nucleotides shorter than the sequence of the exon 1N (164 nucleotides), as the TSS of the later was located at the nucleotide 7457989 of the chromosome 17. Even so, the two variants of the alternative first exon found in the RACEfrags 3 and 4 and the exon 1N share the same 3' end and the same 5'splice site (Figure 3A). Interestingly, the 5' end of exon 1N partially overlaps with the sequence of the novel first exon identified in the RACEfrag 2 (Figure 3A). However, based on our 5'RACE data, the TSS found in the RACEfrag 2, although it is localized further 5'upstream of the TSS described for exon 1N, does not extend the 1N sequence in the 5' end because it uses a different 5' splice site, and, therefore, generates a different 5' exon (Figure 3A).

**Figure 4:**
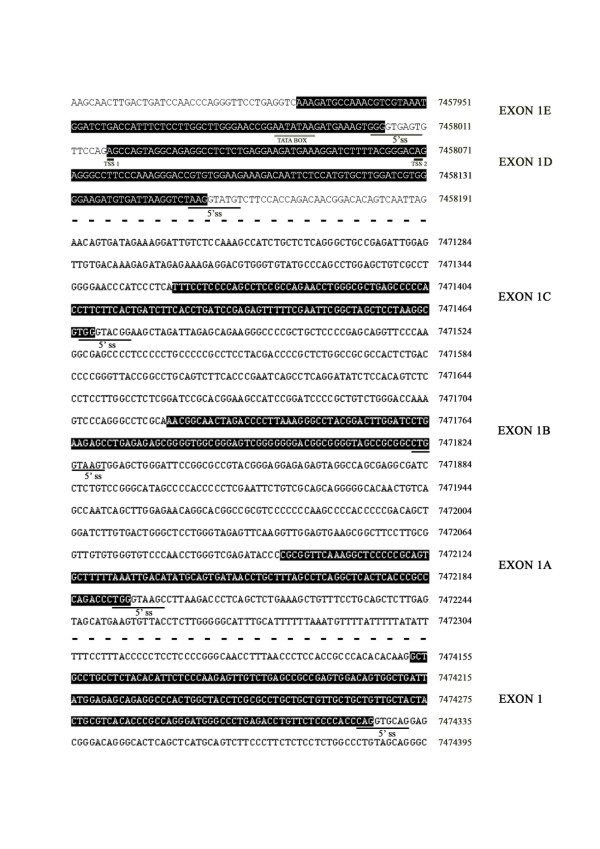
**Localization of SHBG alternative first exons in Chromosome 17 genomic sequence**. Exon sequences (black boxes) and the 5'splice sites (thin underlines) are indicated. The two different TSSs for exon 1D (thick underlines) and the TATA box (gray underline) are also shown. The discontinuities between exon 1D-exon 1C and exon 1A-exon 1 genomic regions are indicated with dashed lines.

Since we have obtained three different alternative first exons in four different RACEfrags, we have named them following their 3' to 5' position in the positive strand of the chromosome 17: exon 1C (RACEfrag 1), exon 1D (RACEfrags 3 and 4) and exon 1E (RACEfrag 2). Exon 1C is found in the chromosome 17 genomic sequence 7471362–7471468, 253 nucleotides upstream of the characterized exon 1B 5' end; exon 1D (previously called exon 1N) is located in the chromosome 17 sequence 7458018/070–7458154, 13.35 kilobases upstream of exon 1C 5' end; and finally, exon 1E is situated just 13 nucleotides upstream from exon 1D TSS (Figure 3B, Figure  4 and Table 2). The analysis of the exon-exon junctions of all RACEfrags sequences, indicated that exons 1C, 1D and 1E were all spliced to exon 2 using the same 3' splice site as exons 1, 1A and 1B (Figure 3C). A complete overview of the characteristics of the different SHBG alternative first exons is illustrated in Table 2.

### Phylogenetic comparison of the SHBG alternative first exons

**Figure 5:**
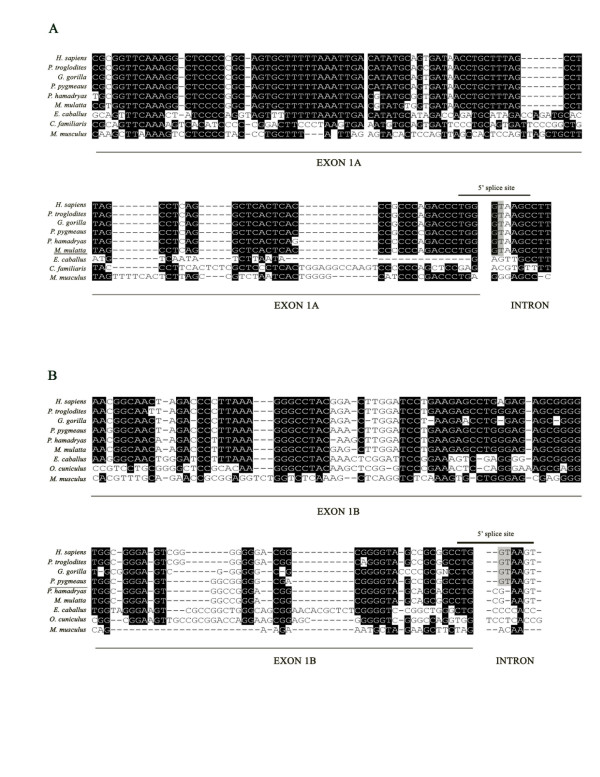
**Phylogenetic comparison of exon 1A and 1B across different vertebrate species**. The sequences of exons 1A (A) and 1B (B) (underlined), the 5' splice sites, composed by the last three nucleotides of the exon and the first five nucleotides of the intron (overlined), and the conserved GT nucleotides of the 5'splice sites (gray overlay) are shown. Last vertebrate specie where the 5'splice site is conserved is underlined.

A search in public databases has led to the identification of the orthologous sequence of exon 1A in the following placental mammals: *Pan troglodytes *(chimpanzee), *Gorilla gorilla *(gorilla), *Pongo pygmaeus *(orangutan), *Papio hamadryas *(Baboon), *Macaca mulatta *(Rhesus macaque), *Equus caballus *(horse), *Canis familiaris *(dog), and *Mus musculus *(mouse). A DNA sequence alignment of exon 1A and the flanking intronic sequence is depicted in Figure 5A. The comparison of apes and Old World monkey sequences showed a 100% sequence identity between humans and chimpanzees, and a calculated 97% sequence identity between humans, orangutans, baboons and rhesus macaques. However, a much less homology is found between humans and horses (calculated sequence identity of 51%); humans and dogs (49%) and humans and mice (44%). The 5'splice site is only conserved in apes and Old World monkeys.

The alignment of the human exon 1B sequence with the sequences of *Pan troglodytes*, *Gorilla gorilla*, *Pongo pygmaeus*, *Papio hamadryas*, *Macaca mulatta*, *Equus caballus*, *Oryctolagus cuniculus *(rabbit), and *Mus musculus *showed a 96% sequence identity between humans and chimpanzees, and a 88–83% sequence identity between humans, gorillas, baboons and rhesus macaques (Figure 5B). Much less homology was found between humans and horses (51%), humans and rabbits (37%) and humans and mice (39%). Exon 1B 5' splice site is only conserved in humans, chimpanzees, gorillas and orangutans, and is not found in the Old World monkeys baboons and rhesus macaques, and neither in horses, rabbits and mice (Figure 5B).

**Figure 6:**
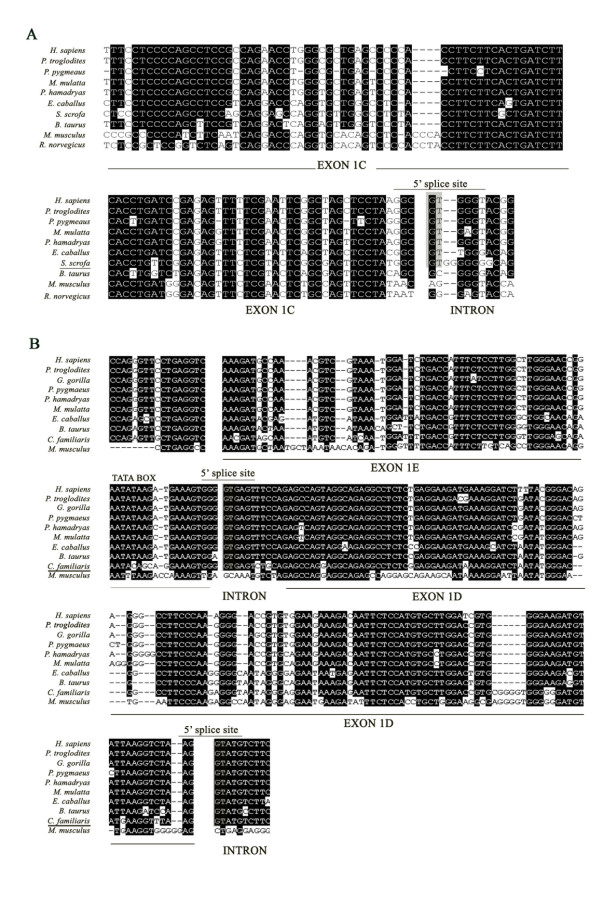
**Phylogenetic comparison of exon 1C, 1 D and 1E across different vertebrate species**. Comparison of the sequences of exons 1C (A), 1 D (B) and 1E (B) (see Figure 5 legend).

The alignment of the human exon 1C sequence with the orthologous sequences of *Pan troglodytes*, *Pongo pygmaeus*, *Papio hamadryas*, *Macaca mulatta*, *Equus caballus*, *Sus scrofa *(pig), *Bos taurus *(bovine), *Mus musculus *and *Rattus norvegicus *(rat) showed a 100% sequence identity with chimpanzees, 90% with orangutans, 95% with baboons, and 96% with rhesus macaques (Figure 6A). There is also a high sequence identity with horses (86%), bovines (79%), pigs (78%), mice (69%) and rats (71%). Therefore, and in contrast to exons 1A and 1B, exon 1C is not only conserved in apes and Old World monkeys, but in horses and pigs. However, it is not conserved in cows, mice and rats.

The alignment of human exon 1D sequence with the orthologous sequences of *Pan troglodytes*, *Gorilla gorilla*, *Pongo pygmaeus*, *Papio hamadryas*, *Macaca mulatta*, *Equus caballus*, *Bos taurus*, *Canis familiaris*, and *Mus musculus *showed a 95% sequence identity with chimpanzees, 96% with gorillas, 94% with orangutans, and 92% with baboons and rhesus macaques. High sequence identity was also found with bovines (82%), horses (81%) and dogs (78%), but less identity was found with mice (57%) (Figure 6B).

Finally, the alignment of human exon 1E sequence with the orthologous sequences of *Pan troglodytes*, *Gorilla gorilla*, *Pongo pygmaeus*, *Papio hamadryas*, *Macaca mulatta*, *Equus caballus*, *Bos taurus*, *Canis familiaris*, and *Mus musculus *showed a 100% sequence identity between humans, chimpanzees and orangutans and a 98% sequence identity between humans, gorillas, baboons and rhesus macaques (Figure 6B). As in the case of exons 1C and 1D, there was a high homology between human and horses, with a calculated sequence identity of 81%. There was also high identity between humans and bovines (80%) and humans and dogs (79%), and less identity was found between humans and mice (62%).

### Activity of the SHBG transcription units in human prostate

Even though the presence of SHBG mRNA in the prostate has been previously reported [15,16], none of these studies took into account the nature of the transcription unit responsible for its production. To analyze the activity of the different SHBG transcription units in prostate tissues and prostate cancer cell lines, we performed RT-PCR using specific forward primers for each of the alternative first exons, as well as an exon 3 forward primer, to determine the global SHBG expression.

**Figure 7:**
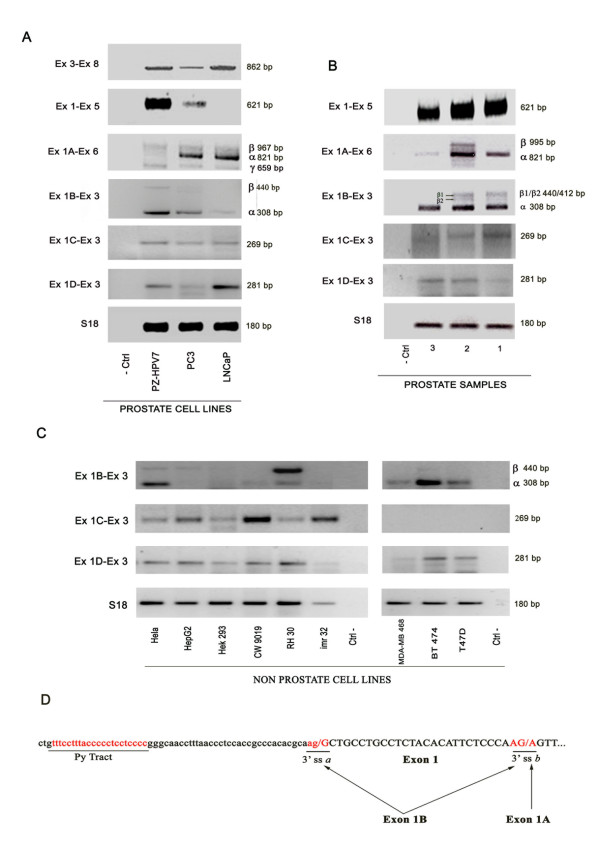
**Identification of the different SHBG transcription units in prostatic and non-prostatic cells by RT-PCR**. A) PCR amplification of transcription units containing exon 1A and 1B in prostate cancer cell lines resulted in two bands: the expected band (α band) derived from exon 1A/1B-exon 2 junction, and an upper band (β band) resulting from the exon 1A/1B-exon 1 junction. A third band, caused by exon 4 skipping (γ band), was also observed in the amplification of 1A transcripts. B) PCR amplification in non-tumoral tissue obtained from human prostate samples. C) PCR amplification in non-prostate cell lines. Negative controls (Ctrl-) contained water instead of cDNA. s18 rRNA was determined to normalize gene expression. D) Scheme of the 5' end of the exon 1 (capital letters) and its flanking 3' intron sequence (small letters). The two 3' splice sites (a and b) are underlined along with the potential pyrimidine tract. The consensus sequences are marked in red.

The analysis of cell lines using a specific exon 3 forward primer, showed that all the prostate cell lines analyzed expressed SHBG, with LNCaP and PZ-HPV-7 cells presenting higher relative amounts of total SHBG mRNA compared with the PC3 cell line (Figure 7A). Using the exon 1 forward primer, the levels of SHBG mRNA were higher in PZ-HPV-7 cells than in PC3 cells, whereas almost no expression was found in LNCaP cells (Figure 7A). On the contrary, when the exon 1A upper primer was used, the levels of SHBG mRNA were higher in LNCaP than in PC3 cells, whereas almost no expression was detected in PZ-HPV-7 cells (Figure 7A). For the exon 1B primer, the maximum levels were found in PZ-HPV7 cells, followed by PC3 cells (Figure 7A), whereas with the exon 1C primer, similar levels of SHBG were found in the three prostate cancer cell lines (Figure 7A). As in the case of exon 3 forward primer, the use of exon 1D forward primer showed that the relative amounts of this transcript were higher in LNCaP and PZ-HPV7 than in PC3 cell line (Figure 7A). In contrast, we could not amplify transcripts containing exon 1E in any of the prostate cell lines tested, probably reflecting that their transcription is very low. It is noteworthy that in exon 1A and exon 1B specific RT-PCR products, a faintly upper-than-expected band was detected (Figure 7A, β band), that once sequenced corresponded to SHBG sequences that included the exon 1 sequence after the exon 1A or exon 1B. These results suggested that exon 1A/exon 1B and exon 1 can be alternatively spliced together and therefore are not always mutually exclusive. When exons 1A or 1B were spliced to exon 2 (Figure 7A, α band), the 5' consensus splice sites of both first exons and the consensus 3' splice site of exon 2 were used (Figure 3C). However, when exons 1A or 1B were spliced to exon 1 (Figure 7A, band β), although the 5' consensus splice sites of exon 1A and 1B were the same, two different 3' splice sites of exon 1 were used, that we named a and b (Figure 7D). While exon 1A was found to be spliced only to the 3' splice site b, exon 1B was alternatively spliced to both a and b splice sites (Figure 7D). Additionally, it was also observed that when exon 1A primers were used, a lower-than-expected band was detected (Figure 7A; γ band), that once sequenced corresponded to SHBG transcripts were exon 1A was spliced to exon 2 and exon 4 was skipped. Using exon 1D primers, a lower-than-expected band was also observed, but in this case, its sequence determined that it corresponded to an unspecific RT-PCR product. No SHBG sequences with non-canonical 5' and 3' consensus splice sites were obtained in any case.

When the activity of the different SHBG transcription units was analyzed in prostate samples, we detected the presence of the ones containing exon 1, 1A, 1B, 1C and 1D in all the samples analyzed (Figure 7B), but exon 1E transcripts were not amplified with RT-PCR. As in the case of the prostate cancer cell lines, upper-than-expected RT-PCR bands were detected (Figure 7B, β band) that, once sequenced, corresponded to specific SHBG transcripts whose exon 1A and 1B sequences were followed by the exon 1 sequence using the 3' splice sites a and b (Figure 7D). In the case of exon 1B transcripts, two different β bands were observed (Figure 7B; β_1 _and β_2 _bands) corresponding to the use of the 3' splice sites a (β_1_) and b (β_2_).

**Figure 8:**
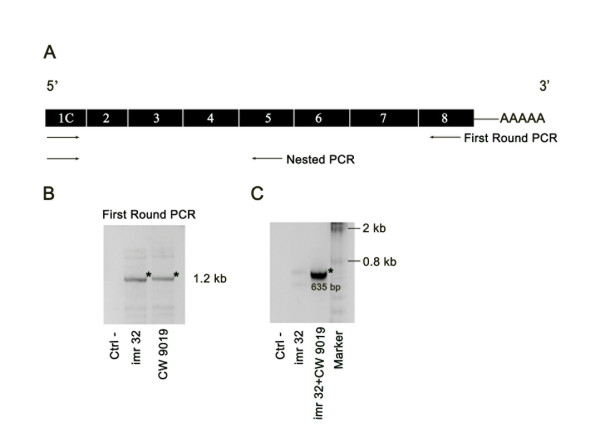
**Amplification of the full-length exon 1C SHBG transcript**. A) Scheme of the nested PCR designed to amplify the full-length exon 1C transcript from cDNA of *imr *32 and CW 9019 cell lines, using primers directed against exons 1C and 8 (first round) and against exons 1C and 5 (second round). B) A major 1.2 kb band (asterisk) was obtained in the first round. C) A 635 bp PCR product resulted from the second round (asterisk). The direct sequencing of this product confirmed the SHBG exon 1C sequence. *imr *32: cDNA of *imr *32 cells used as positive control. Ctrl-: negative control using water instead of cDNA.

To determine whether transcription of these novel SHBG isoforms was restricted to prostate, human non-prostatic cell lines were analyzed by RT-PCR. The results revealed that transcripts containing exon 1B were present in cell lines derived from cervix carcinoma (HeLa), rhabdomyosarcoma (CW 9019 and RH 30), and breast cancer (MDA-MB 468, T47D, and BT 474) (Figure 7C), but were almost undetectable in the hepatocarcinoma cell line HepG2, in the kidney cell line Hek 293, and in the neuroblastoma cell line *imr *32 (Figure 7C). As in the case of prostate cell lines and tissues, the upper-than-expected band corresponding to transcripts where exon 1B is followed by the exon 1 sequence, was also detected in the RH 30, HeLa and HepG2 cell lines. Specifically, in the RH 30 cell line, transcripts containing exon 1B-exon 1 were more abundant than exon 1B-exon 2 transcripts (Figure 7C). The transcription unit containing exon 1C was identified in HeLa, HepG2, Hek 293, CW 9019, RH 30 and *imr *32 cells, but no detectable levels were found in any of the breast cancer cell lines analyzed (Figure 7C). Furthermore, in cell lines where levels of exon 1C SHBG mRNA were higher (CW 9019 and *imr *32; Figure 7A), full length exon 1C transcript sequence was detected by nested PCR, using primers directed against exons 1C – 8 for the first round, and exons 1C – 5 for the second round (Figure 8A). The major product found in the first round (1.2 kb), coincided with the expected size of the full-length transcript from exon 1C to exon 8 (Figure 8B). The 1.2 kb PCR band from both cell lines were excised from the gel, purified and pooled to use it as template DNA for the second round PCR. Direct sequencing of the second round PCR product demonstrated that the 635 nucleotide sequence contained the exon 1C followed by exons 2, 3, 4 and 5 (Figure 8C). Finally, the transcription unit of exon 1D was detected in all cell lines analyzed, but very few levels were found in the *imr *32 and MDA-MB 468 cells (Figure 7C). The full-length of transcripts beginning with exon 1D has been previously reported by Kahn and collaborators as exon 1N SHBG transcript [GenBank: EU352670].

The analysis of the relative abundance of the alternative 1B, 1C and 1D transcripts compared to 1A in LNCaP, PC3 and PZ-HPV7 prostate cell lines as well as in HeLa cells by real-time PCR showed that the levels of exon 1B transcript were significantly higher in PC3 and PZ-HPV7 cells, and of exon 1D transcript in PZ-HPV7 cells (see Additional file 1).

## Discussion

Recent reports in the framework of the ENCODE Project indicated that more than two thirds of the interrogated genes present additional TSSs upstream of their annotated first exons [18]. The analysis of 44 regions totaling 30 Mb or 1% of the human genome sequence showed that there is a coding protein gene every 62 kb, and that the novel TSSs often mapped upstream loci, since the newly identified TSSs localized on average 186 kb upstream of the most 5' annotated exons [18,24]. Additionally, an increasing number of reports indicate that many eukaryotic genes possess multiple transcriptional promoters associated with alternative first exons [25-28]. In this context, we decided to characterize the 5' end of the human SHBG gene.

In chromosome 17, where SHBG is localized, alternative splicing has been described to occur extensively, with an average of 5 transcripts per gene, and it has also been shown that in this chromosome, 76.6% of the genes display, at least, two transcripts [29]. In the present study, we have used *in silico *and *in vitro *approaches in order to identify novel 5' SHBG alternative first exons. Using the FirstEF program we identified a potential novel SHBG first exon in the positive strand of chromosome 17, situated -278 nucleotides upstream of the TSS of the SHBG alternative exon 1. We named this potential novel SHBG first exon, exon 1B, and therefore we renamed the previously described alternative exon 1 [23] as exon 1A. Using RT-PCR, we confirmed the presence of SHBG exon 1B transcripts in prostate tissues and cell lines. The length of exon 1B was 104 nucleotides (7471721–7471824), but a 5' extension can not be excluded, since its TSS has not been identified yet. Exon 1B overlaps with the nucleotides 169–66 of the 5'UTR sequence of the SAT2 gene [GenBank: NM_133491.2]. As the SAT2 gene is situated in the negative strand of the chromosome 17, SHBG and SAT2 genes would produce natural sense-antisense pair transcripts that overlap head to head. Moreover, the analysis of the SHBG 5'genomic sequence with the FirstEF program indicates that exon 1B is contained in a CpG island of 202 nucleotides of length (7471655–7471855). In this regard, it has been estimated that over 20% of human transcripts might form sense-antisense pairs [30] and also that CpG island promoter regions are commonly associated with bidirectional promoter activity (approximately 15% of the imprinted genes have associated antisense transcripts) [31,32]. One example is the Gabpa-Atp5j genes in mouse, which contain two over-lapping promoters in each direction [33]. When this condition occurs in yeast and bacteria, there is potential for transcriptional interference [34], and it has also been postulated that, in these CpG bidirectional promoters, the activity of one promoter might influence the epigenetic state of the other [33].

Using 5'RLM-RACE with cDNA obtained from DU-145 and LNCaP cells, we identified two additional SHBG first exons that were named exons 1C and 1E, and two different sequences corresponding to the previously described exon 1N. These two sequences resulted from two different TSSs, generating exon sequences 27 and 79 nucleotides shorter than exon 1N. We renamed these exon sequences as exon 1D.

Exon 1C has a length of 107 nucleotides and is localized 253 nucleotides upstream of exon 1B. As in the case of exon 1B, it also overlaps with the SAT 2 gene, specifically with the complete coding sequence of exon 3 and with 44 and 10 nucleotides of intron 3 and intron 2 respectively. The TSS identified by 5'RACE, with T as the nucleotide +1, is displaced just one nucleotide from a consensus Inr element or Cap motif (pyrimidine, pyrimidine, A(+1), N, T/A, pyrimidine, pyrimidine, where N is any nucleotide)[33]. Exon 1D has a length of 137 or 85 nucleotides, depending on which TSS is used, and is localized 13.35 kilobases 5' upstream of exon 1 TSS. Exon 1E has a length of 75 nucleotides and its 3' end is situated only 13 nucleotides upstream of the TSS1 of exon 1D. It overlaps with the intron 1 sequence of the FXR2 gene [GenBank: NM_004860.2], situated also in the negative strand. This overlap was previously described in rat chromosome 10, where a GC rich sequence in the alternative promoter of rat SHBG overlapped with the 5' UTR of the FXR2 gene [35]. These and our results, suggest that the 5' end of the SAT2 and FXR2 genes present a broad range of TSSs in the opposite strand, corresponding to the SHBG gene.

TATA boxes have been described to be six-fold more common in genes with single promoters than in genes with alternative promoters, and it has been suggested that strong TATA boxes might be incompatible with alternative promoters [36]. However, in exon 1E there is a TATAA sequence situated 30 nucleotides upstream from the TSS1 of exon 1D, probably regulating its transcription initiation. TATA-box directed transcription is normally associated with a sharply defined TSS situated 30–31 downstream from the TATA sequence. In contrast, TATA-independent promoters normally present a much broad distribution of TSSs [33], suggesting that exons 1B, 1C and 1E might present additional TSSs than the ones presented here. Furthermore, while TATA-box promoters are normally associated with tightly regulated transcripts with a strong bias toward postnatal activity [33,36], broad TSS promoters and CpG promoters are associated with ubiquitous transcripts, and, in the case of genes with alternative promoters, with a weak bias toward prenatal transcription [33,36]. These data suggests a differential usage of the SHBG alternative promoters through different stages of development.

The full-length of transcripts beginning with exon 1B and 1C has been demonstrated. In the case of exon 1C transcript, although the size of the product of the first round PCR suggests that exons 6 and 7 are included in the full-length transcript, it has only been possible to sequence it from exon 1C to exon 5.

We have shown the presence of exon 1B, 1C and 1D transcripts in prostate cell lines and tissues as well as in several non-prostatic cell lines. These results support that the activity of these different transcription units is not restricted to prostate, as it would be expected for 1B and 1C transcripts, since their promoters do not contain a TATA sequence. As for the 1D transcripts, the activity of the putative TATA promoter remains to be proven. With regard to the relative abundance of the alternative transcripts, real-time PCR assays showed that the highest levels corresponded to the exon 1B transcript in the four cell lines analyzed. Although differences in primer efficiency could not be completely excluded, it would be interesting in the future to elucidate the meaning of these variations.

All the SHBG exons identified by 5'RACE in this study are very well defined in their 3' end by consensus splice sites sequences (AG/GTRAGT), and they are all spliced directly to the 3'splice site of exon 2, as it was previously described for SHBG exon 1 and exon 1A [9]. However, when RT-PCR was performed in prostate cancer cell lines and prostate tissues using specific primers for exons 1A and 1B, two different 3'splice sites of exon 1 were used, a and b, both presenting a consensus -AG/G sequence for U2AF65 protein binding, but lacking a clear polypyrimidine tract and branch point. None of this alternative first exons contain in their sequence an ATG in frame with the SHBG coding sequence, suggesting that they act as 5' UTR sequences regulating the translation efficiency from the first ATG in frame of exon 2, which encodes the methionine 30 of the transcripts that begin with exon 1. In this regard, it has been reported that stable mRNA secondary structures in the 5'UTR region (≥-35 kcal/mol) can decrease considerably the translation efficiency by affecting ribosomal recruitment and positioning at the initiation codon [37]. The MFOLD program served us to predict a hairpin stability ≥-40 kcal/mol for exons 1A, 1B, 1C and 1D (TSS1), and -35,8 kcal/mol for exon 1E, supporting that the secondary structure of the different alternative first exons could affect translation efficiency.

The functional significance of these novel alternative SHBG transcription units will rely on the demonstration of their protein coding capacity or of their action as natural antisense transcripts of genes located on the opposite DNA strand. We tested the former possibility by using different antibodies against SHBG, and detected bands of the expected size in human prostate samples but not in prostate cancer cell lines by Western blot analysis (data not shown). Our preliminary data of transient transfection experiments of different alternative SHBG full-length constructs showed that these transcripts are indeed translated, but their translation efficiency are negatively regulated depending on each specific 5'UTR sequence, as predicted by the MFOLD program. Further studies are required to fully assess and understand the contribution of the use of these alternative transcripts and their probable alternative promoters to regulate transcription and translation, as it has already been demonstrated for other genes [28]. As for the second possible action, the discovery of novel 5'SHBG exons that overlap with the SAT 2 and FXR 2 sequences (exons 1B, 1C, 1D and 1E) suggests that SHBG-SAT 2 and SHBG-FXR 2 genes might be mutually regulated by transcriptional interference.

The phylogenetic comparison of the human SHBG alternative first exons with different vertebrate mammalian species showed that exons 1C, 1D and 1E are highly conserved across the species, but exon 1A and specially exon 1B, are by far less conserved. The variation in the degree of conservation of the different SHBG alternative first exons parallels the degree of conservation of their 5' splice sites: while the 5'splice site of exons 1A and 1B are only conserved within primate species, the ones of exons 1C, 1D and 1E are conserved in primate and non-primate species. These data suggest that 1A and 1B are recently evolved exons, with higher evolutionary turnover rate, especially exon 1B, which was created about 25 million years ago, when apes diverged from Old World monkeys.

The estimation of the total number of human protein-coding genes falls between 20000–25000 [38,39], whereas those of simpler organisms as *Drosophila melanogaster *and *Caenorhabditis elegans *are not much lower, with 13000 and 18000 genes respectively [38]. It was hypothesized that functional diversity of this limited number of genes is necessary to create the highly elaborated systems necessary for mammalian live [38]. Alternative splicing and alternative promoter usage are well-described mechanisms that produce an elevated number of protein-coding and non-coding transcripts from a single gene locus. In the framework of the ENCODE project it was observed that 86% of the interrogated multi-exon gene loci in the ENCODE regions presented alternative splicing generating > 5.4 transcripts per gene [18]. Our analysis showed that, at least, one additional transcription unit (exon 1B) aroused in apes, due to a single nucleotide change that generated a new 5' splice site in exon 1B.

## Conclusion

In the present study, we have identified three novel alternative SHBG first exons (exons 1B, 1C and 1E), and further characterized an alternative SHBG first exon previously introduced in the public databases (exon 1D). We have also demonstrated the activity of the transcription units containing exons 1B, 1C and 1D in human prostate tissues and prostatic and non-prostatic cell lines. In view of these results, it will be necessary to determine the significance of these alternative TSSs in terms of regulation of expression of SHBG or overlapping genes on the opposite strand. Additionally, it would be interesting, in the future, to ascertain whether the appearance of a SHBG alternative transcript in humans confers any evolutionary advantage.

## Authors' contributions

TP and ABD carried out the major part of the experiments and data analysis. FM conceived, designed and coordinated the study. TP and FM write the manuscript. AH and AS performed part of the experiments. IdT contributed to the material acquisition and histological identification. JM and JR contributed to the project design. All authors participated in data interpretation and revised and approved the final manuscript.

## Supplementary Material

Additional file 1 **Relative abundance of the alternative SHBG transcripts in cell lines by real-time PCR**. The data represent the means ± SE for each SHBG alternative exon in relation to the levels of alternative exon 1A, using one-way ANOVA. Click here for file

## References

[B1] Hammond GL (1995). Potential functions of plasma steroid-binding proteins. Trends Endocrinol Metab.

[B2] Berube D, Seralini GE, Gagne R, Hammond GL (1990). Localization of the human sex hormone-binding globulin gene (SHBG) to the short arm of chromosome 17 (17p12–p13). Cytogenet Cell Genet.

[B3] Cousin P, Billotte J, Chaubert P, Shaw P (2000). Physical map of 17p13 and the genes adjacent to p53. Genomics.

[B4] Selva DM, Hogeveen KN, Seguchi K, Tekpetey F, Hammond GL (2002). A human sex hormone-binding globulin isoform accumulates in the acrosome during spermatogenesis. J Biol Chem.

[B5] Janne M, Hammond GL (1998). Hepatocyte nuclear factor-4 controls transcription from a TATA-less human sex hormone-binding globulin gene promoter. J Biol Chem.

[B6] Selva DM, Bassas L, Munell F, Mata A, Tekpetey F, Lewis JG, Hammond GL (2005). Human sperm sex hormone-binding globulin isoform: characterization and measurement by time-resolved fluorescence immunoassay. J Clin Endocrinol Metab.

[B7] Selva DM, Hammond GL (2006). Human sex hormone-binding globulin is expressed in testicular germ cells and not in sertoli cells. Horm Metab Res.

[B8] Forges T, Gerard A, Hess K, Monnier-Barbarino P, Gerard H (2004). Expression of sex hormone-binding globulin (SHBG) in human granulosa-lutein cells. Mol Cell Endocrinol.

[B9] Hammond GL, Underhill DA, Rykse HM, Smith CL (1989). The human sex hormone-binding globulin gene contains exons for androgen-binding protein and two other testicular messenger RNAs. Mol Endocrinol.

[B10] Hryb DJ, Khan MS, Romas NA, Rosner W (1989). Solubilization and partial characterization of the sex hormone-binding globulin receptor from human prostate. J Biol Chem.

[B11] Moore KH, Bertram KA, Gomez RR, Styner MJ, Matej LA (1996). Sex hormone binding globulin mRNA in human breast cancer: detection in cell lines and tumor samples. J Steroid Biochem Mol Biol.

[B12] Noe G (1999). Sex hormone binding globulin expression and colocalization with estrogen receptor in the human Fallopian tube. J Steroid Biochem Mol Biol.

[B13] Larrea F, Diaz L, Carino C, Larriva-Sahd J, Carrillo L, Orozco H, Ulloa-Aguirre A (1993). Evidence that human placenta is a site of sex hormone-binding globulin gene expression. J Steroid Biochem Mol Biol.

[B14] Schock HW, Herbert Z, Sigusch H, Figulla HR, Jirikowski GF, Lotze U (2006). Expression of androgen-binding protein (ABP) in human cardiac myocytes. Horm Metab Res.

[B15] Hryb DJ, Nakhla AM, Kahn SM, St George J, Levy NC, Romas NA, Rosner W (2002). Sex hormone-binding globulin in the human prostate is locally synthesized and may act as an autocrine/paracrine effector. J Biol Chem.

[B16] Kahn SM, Hryb DJ, Nakhla AM, Romas NA, Rosner W (2002). Sex hormone-binding globulin is synthesized in target cells. J Endocrinol.

[B17] Ng KM, Catalano MG, Pinos T, Selva DM, Avvakumov GV, Munell F, Hammond GL (2006). Evidence that fibulin family members contribute to the steroid-dependent extravascular sequestration of sex hormone-binding globulin. J Biol Chem.

[B18] Denoeud F, Kapranov P, Ucla C, Frankish A, Castelo R, Drenkow J, Lagarde J, Alioto T, Manzano C, Chrast J (2007). Prominent use of distal 5' transcription start sites and discovery of a large number of additional exons in ENCODE regions. Genome Res.

[B19] Davuluri RV, Grosse I, Zhang MQ (2001). Computational identification of promoters and first exons in the human genome. Nat Genet.

[B20] Cartegni L, Wang J, Zhu Z, Zhang MQ, Krainer AR (2003). ESEfinder: A web resource to identify exonic splicing enhancers. Nucleic Acids Res.

[B21] Fairbrother WG, Yeo GW, Yeh R, Goldstein P, Mawson M, Sharp PA, Burge CB (2004). RESCUE-ESE identifies candidate exonic splicing enhancers in vertebrate exons. Nucleic Acids Res.

[B22] Zuker M (2003). Mfold web server for nucleic acid folding and hybridization prediction. Nucleic Acids Res.

[B23] Gershagen S, Lundwall A, Fernlund P (1989). Characterization of the human sex hormone binding globulin (SHBG) gene and demonstration of two transcripts in both liver and testis. Nucleic Acids Res.

[B24] (2004). The ENCODE (ENCyclopedia Of DNA Elements) Project. Science.

[B25] Kim TH, Barrera LO, Qu C, Van Calcar S, Trinklein ND, Cooper SJ, Luna RM, Glass CK, Rosenfeld MG, Myers RM (2005). Direct isolation and identification of promoters in the human genome. Genome Res.

[B26] Cooper SJ, Trinklein ND, Anton ED, Nguyen L, Myers RM (2006). Comprehensive analysis of transcriptional promoter structure and function in 1% of the human genome. Genome Res.

[B27] Tan JS, Mohandas N, Conboy JG (2006). High frequency of alternative first exons in erythroid genes suggests a critical role in regulating gene function. Blood.

[B28] Davuluri RV, Suzuki Y, Sugano S, Plass C, Huang TH (2008). The functional consequences of alternative promoter use in mammalian genomes. Trends Genet.

[B29] Zody MC, Garber M, Adams DJ, Sharpe T, Harrow J, Lupski JR, Nicholson C, Searle SM, Wilming L, Young SK (2006). DNA sequence of human chromosome 17 and analysis of rearrangement in the human lineage. Nature.

[B30] Chen J, Sun M, Kent WJ, Huang X, Xie H, Wang W, Zhou G, Shi RZ, Rowley JD (2004). Over 20% of human transcripts might form sense-antisense pairs. Nucleic Acids Res.

[B31] Lehner B, Williams G, Campbell RD, Sanderson CM (2002). Antisense transcripts in the human genome. Trends Genet.

[B32] Trinklein ND, Aldred SF, Hartman SJ, Schroeder DI, Otillar RP, Myers RM (2004). An abundance of bidirectional promoters in the human genome. Genome Res.

[B33] Carninci P, Sandelin A, Lenhard B, Katayama S, Shimokawa K, Ponjavic J, Semple CA, Taylor MS, Engstrom PG, Frith MC (2006). Genome-wide analysis of mammalian promoter architecture and evolution. Nat Genet.

[B34] Prescott EM, Proudfoot NJ (2002). Transcriptional collision between convergent genes in budding yeast. Proc Natl Acad Sci USA.

[B35] Joseph DR (1998). The rat androgen-binding protein (ABP/SHBG) gene contains triplet repeats similar to unstable triplets: evidence that the ABP/SHBG and the fragile X-related 2 genes overlap. Steroids.

[B36] Baek D, Davis C, Ewing B, Gordon D, Green P (2007). Characterization and predictive discovery of evolutionarily conserved mammalian alternative promoters. Genome Res.

[B37] Babendure JR, Babendure JL, Ding JH, Tsien RY (2006). Control of mammalian translation by mRNA structure near caps. Rna.

[B38] Lander ES, Linton LM, Birren B, Nusbaum C, Zody MC, Baldwin J, Devon K, Dewar K, Doyle M, FitzHugh W (2001). Initial sequencing and analysis of the human genome. Nature.

[B39] Venter JC, Adams MD, Myers EW, Li PW, Mural RJ, Sutton GG, Smith HO, Yandell M, Evans CA, Holt RA (2001). The sequence of the human genome. Science.

