# Supplementary material for: Identification, characterization and expression of novel Sex Hormone Binding Globulin alternative first exons in the human prostate
Source: BMC Mol Biol. 2009 Jun 17;10:59. doi: 10.1186/1471-2199-10-59 (PMC2706245; doi:10.1186/1471-2199-10-59)
Supplement: Additional file 1 — Relative abundance of the alternative SHBG transcripts in cell lines by real-time PCR. The data represent the means ± SE for each SHBG alternative exon in relation to the levels of alternative exon 1A, using one-way ANOVA. [file 1471-2199-10-59-S1.pdf]

Cell line	Exon	Mean ± SE	P value
LNCaP	1B	2.13 ± 0.88	p< 0.2919
	1C	0.84 ± 0.36	p< 0.6568
	1D	1.12 ± 0.56	p< 0.8944
PC3	1B	15.07 ± 1.08	p< 0.0003
	1C	3.07 ± 0.88	p< 0.1346
	1D	0.59 ± 0.16	p< 0.3026
PZ-HPV7	1B	99.33 ± 10.16	p< 0.0006
	1C	9.44 ± 4.40	p< 0.1276
	1D	3.08 ± 0.67	p< 0.0379
HeLa	1B	11.96 ± 5.92	p< 0.1391
	1C	0.99 ± 0.41	p< 0.9109
	1D	0.88 ± 0.37	p< 0.7156
